# Reduction of Phantom Limb Pain and Improved Proprioception through a TSR-Based Surgical Technique: A Case Series of Four Patients with Lower Limb Amputation

**DOI:** 10.3390/jcm10174029

**Published:** 2021-09-06

**Authors:** Alexander Gardetto, Eva-Maria Baur, Cosima Prahm, Vinzenz Smekal, Johannes Jeschke, Gerfried Peternell, Michael T. Pedrini, Jonas Kolbenschlag

**Affiliations:** 1 Competence Center for Bionic Prosthetics, Department of Plastic, Aesthetic and Reconstructive Surgery with Hand Surgery, Brixsana Private Clinic, 39042 Bressanone, Italy; 2 Department of Plastic, Aesthetic and Reconstructive Surgery, Medical University of Innsbruck, 6020 Innsbruck, Austria; eva-maria.baur@tirol-kliniken.at; 3 BG Trauma Center Tuebingen, Department of Hand, Plastic, Reconstructive and Burn Surgery, Eberhard Karls University Tuebingen, 72108 Tuebingen, Germany; cprahm@bgu-tuebingen.de (C.P.); jonaskolbenschlag@gmail.com (J.K.); 4 AUVA Trauma Center Klagenfurt, Department of Trauma Surgery, 9020 Klagenfurt, Austria; Vinzenz.Smekal@auva.at; 5 Department of Plastic, Aesthetic and Reconstructive Surgery, Maria Hilf Private Clinic, 9020 Klagenfurt, Austria; johannes.jeschke@humanomed.at; 6 Department of Exoprosthetics, AUVA Rehabilitation Clinic, 8144 Tobelbad, Austria; Gerfried.Peternell@auva.at; 7 Department of Internal Medicine, Brixsana Private Clinic, 39042 Bressanone, Italy; michael.pedrini@brixsana.it

**Keywords:** phantom limb pain, neuroma pain, amputation, lower extremity, targeted sensory reinnervation, TSR

## Abstract

Four patients underwent targeted sensory reinnervation (TSR), a surgical technique in which a defined skin area is first selectively denervated and then surgically reinnervated by another sensory nerve. In our case, either the area of the lateral femoral cutaneous nerve or the saphenous nerve was reinnervated by the sural nerve. Patients were then fitted with a special prosthetic device capable of transferring the sense of pressure from the sole of the prosthesis to the newly wired skin area. Pain reduction after TSR was highly significant in all patients. In three patients, permanent pain medication could even be discontinued, in one patient the pain medication has been significantly reduced. Two of the four patients were completely pain-free after the surgical intervention. Surgical rewiring of existing sensory nerves by TSR can provide the brain with new afferent signals seeming to originate from the missing limb. These signals help to reduce phantom limb pain and to restore a more normal body image. In combination with special prosthetic devices, the amputee can be provided with sensory feedback from the prosthesis, thus improving gait and balance.

## 1. Introduction

Phantom limp pain (PLP) and neuroma pain (NP) are highly prevalent in amputees and severely reduce their quality of life. PLP after amputation is based on multifactorial mechanisms and is present in 60 to 90% of patients [1,2,3]. NP, caused by terminal neuromas, occurs in 13–32% of amputees and compromises not only quality of life but also prosthetic tolerance and functional independence [4,5]. As of today, there is no gold standard of treatment for either PLP or NP. Moreover, most therapies are purely symptomatic in nature, such as pain medication, nerve blocks, or neurostimulators [3,6,7,8,9]. Loss of information from the missing limb is one of the key factors in the development of PLP [2]. Thus, recreating a pathway for such information and therefore “reinstalling” the limb within the brain might be a central and causal step in the treatment of PLP. Such a pathway can be created surgically by the rearrangement of motor and sensory nerves [10]. Experience with the transfer of motor nerves to improve prosthetic control in the upper extremity (targeted muscle reinnervation, TMR) exists for over a decade [11,12,13]. Interestingly, these patients with nerves redirected to their chest muscles were able to feel their hand on their chest wall, pointing towards the possibility of targeted sensory reinnervation (TSR) [14]. TSR of residual nerves in the residual upper limb has been shown to create authentic sensations from the missing limb. Such a sensory feedback is considered to be important for further improvement of prosthetic control but also for the reduction of phantom pain [10,15,16,17,18,19]. There are few reports on the application of sensory feedback systems for prosthesis on the lower limb [20,21,22]. In particular, to our knowledge, no such techniques for future interaction between TSR and prostheses equipped with a sensory feedback system have been published. Some surgical approaches to restore sensation have been introduced over the last years to treat or prevent post-amputation pain in the lower limb [23,24]. These two reports are based on implanted electrodes to stimulate the residual sciatic nerve with feedback from the prosthesis. However, such implanted electrodes are invasive, costly, and induce scarring of the nerve. Therefore, creation of an easily accessible and purely autologous human–machine interface for tactile feedback seems highly desirable. In this case series, we describe a surgical method based on TSR fulfilling these criteria. Our method is able to give back the authentic feeling of the foot when combining it with the medical product “Suralis”, a vibrotactile feedback system developed by Saphenus Medical Technology. This combination reduces and even prevents PLP as well as NP and improves prosthetic rehabilitation.

## 2. Materials and Methods

After obtaining approval from the local ethics committee (no. M2020-37), a retrospective chart review was conducted. All patients undergoing TSR on the lower extremity at our institutions from October 2014 to December 2020 were identified. Patients who underwent surgeries to the residual limb after TSR for non-pain related reasons were excluded from the study. Three patients had amputations due to trauma, one patient due to fulminant venous thrombosis of the leg. In three patients, the indication for surgical intervention was treatment-resistant PLP and NP. In one patient, it was performed prophylactically. Contraindications for surgery were an injured skin area for reinnervation at the lateral site of the femoral stump or at the medial part of the lower leg stump, as well as an injured or missing sural nerve.

### 2.1. Patients

Patient 1 suffered a stroke in April 2007 at the age of 46. A fulminant deep vein thrombosis that developed during treatment of the stroke led to an amputation of his lower left leg. Soon after amputation, the patient developed severe PLP and NP. Despite opiates and psychopharmaceuticals as well as conservative therapy, the pain could not be sufficiently managed. Thus, he underwent TSR on his transtibial stump in 2014.

Patient 2 had a motorcycle accident in May 1989 at the age of 25, resulting in an open comminuted femur fracture and rupture of the left popliteal artery, open comminuted fracture of the left lower leg, bilateral radius and ulna fracture and fracture of the fifth cervical vertebra. The patient developed a compartment syndrome in his lower left leg, osteomyelitis of the tibia and three consecutive pulmonary embolisms. The patient had a total of 35 surgical interventions with numerous attempts for reconstruction of his left lower extremity. Despite these efforts, a trans-articular amputation was performed in September 2005. Due to recurrent osteomyelitis, four revisions of the stump were performed resulting in transfemoral amputation. The patient developed intense PLP and NP, reducing quality of life to such an extent, that the patient often had to struggle with suicidal ideation. This pain was not controllable by medication despite maximal doses of multiple agents. Also, the patient was unable to wear the prosthesis for more than a few hours and especially its donning and doffing was particularly painful. In 2018, it was decided to perform TSR surgery on the thigh. 

Patient 3 was involved in a car accident in 2017 at the age of 42 during which he suffered extensive injuries, including an open femoral fracture on the left side. The patient underwent a total of 12 surgeries on this extremity, including nail arthrodesis of the knee joint. Unfortunately, boney union was not achieved, rendering mobilization impossible without severe pain and mobility aids. In addition, he developed severe NP which could not be sufficiently managed conservatively. Taking all things into consideration and after several consultations and under psychological supervision, the patient decided to undergo transfemoral amputation with simultaneous prophylactic TSR surgery in the autumn of 2019.

Patient 4 had a motorcycle accident in 2011 at the age of 50 and suffered a subtotal transtibial amputation on her left and a fracture of the left femur. Due to the severe injury, her lower leg had to be amputated just below the knee at the day of the accident. The patient underwent a total of six surgeries, including two to treat neuromas in 2015 and 2016. Despite these efforts, she developed severe PLP and NP, uncontrollable by medication and conservative therapy. After several consultations, she underwent TSR surgery in 2020. A summary of these patients is given in Table 1. 

### 2.2. Procedures

Patients were only considered for TSR surgery when they suffered life-altering pain despite a maximum of conservative treatment. All patients were treated by a specialized pain physician and assessed by a psychologist before surgery. 

### 2.3. Assessments

Prior to surgery, all patients were subjected to a thorough examination, including neurological and high-resolution ultrasound examinations, to assess the state of existing neuroma. Pain scores (Numeric Rating Scale (NRS) and Visual Analog Scale (VAS)) were also assessed. Patient’s quality of life after the procedure was evaluated with the SF-36 health questionnaire (SF-36). The SF-36 measures health status in eight dimensions: physical functioning (PF), role limitations due to physical health problems (RP); role limitations due to emotional problems (RE); bodily pain (BP); general health perceptions (GH); vitality (VT); social functioning (SF); and general mental health (MH). Based on these eight subscales, two superior physical and mental component summary scales are calculated. The outcome scores were compared with age- and sex-matched samples of the norm population, a lower limb amputee population, and a population experiencing longstanding illness. Post-operatively, all patients were followed-up routinely via out-patient visits and by phone. During out-patient visits, “sensory mappings” were drawn to depict the re-innervation of the skin area and where the patients sensed their foot within this area. The last patient also underwent functional assessments with a specialized prosthetic device, including gait pattern analysis, four square step test, timed up and go test and 6 min walk test [25,26,27].

### 2.4. Analysis

We applied a non-parametric test to compare the matched samples of measurements from all subtests with different underlying distributions, each tested at two different time points: before and after the application of the Suralis device (Saphenus Medical Technology, 3500 Krems, Austria) using a conventional prosthesis as control. 

### 2.5. Surgical Techniques

The surgical technique depends on the level of the amputation because it delineates which nerves and skin areas are available for TSR surgery. The common goal is to reinnervate a dedicated skin area via the sural nerve, thus enabling the patient to genuinely feel the former autonomous area of the sural nerve on their residual limb.

**Figure 1:**
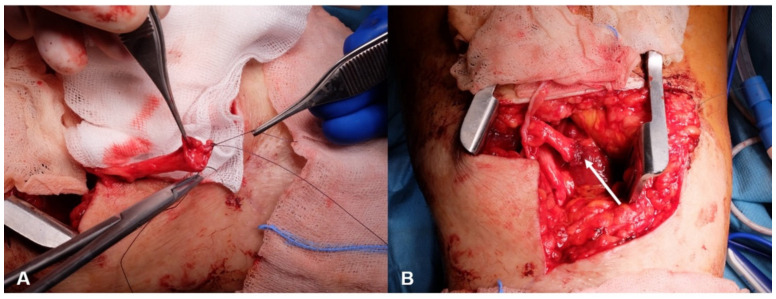
Prevention of neuroma recurrence: (**A**) nerve stump covering with an epineural cap; (**B**) transposition of this nerve stump into muscle (arrow).

In a transtibial amputee (such as patient 1), the recipient of the sensory transfer is the saphenous nerve with its autonomous skin area on the medial, proximal lower leg. First, the neuroma of the tibial nerve and common peroneal nerve stumps are identified and resected. To prevent neuroma recurrence, nerve stumps are covered with an epineural cap and transposed into muscle (Figure 1).

**Figure 2:**
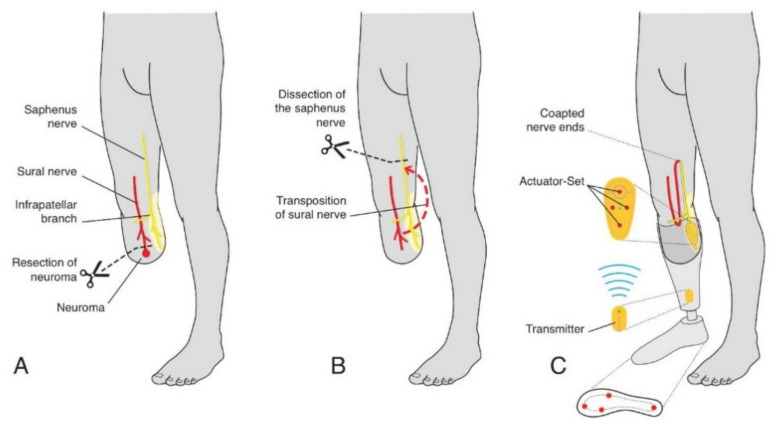
Illustration of the surgical technique for a transtibial amputation: (**A**) resection of the stump neuroma; (**B**) transposition of the ipsilateral sural nerve to the cutaneous territory of the saphenous nerve; (**C**) medical device Suralis in place, providing sensory feedback from the sole to the reinnervated skin area.

Then, the TSR surgery per se is carried out by first identifying the saphenous nerve above the knee. Next, the sural nerve is identified, its end neuroma resected, and the nerve is dissected proximally. It is important to mobilize the nerve sufficiently to allow its transposition to the medial side of the upper leg via a sub-cutaneous tunnel. Then, the saphenous nerve is transected proximally to allow tension-free end-to-end coaptation with the sural nerve (Figure 2). The proximal stump of the saphenous nerve is embedded into muscle tissue of the adductors for neuroma prophylaxis. 

For transfemoral amputees, the recipient for TSR is the lateral femoral cutaneous nerve with its autonomous skin territory on the lateral thigh. Due to the distance between the nerve ends, a sural nerve graft from the contralateral leg is necessary. 

**Figure 3:**
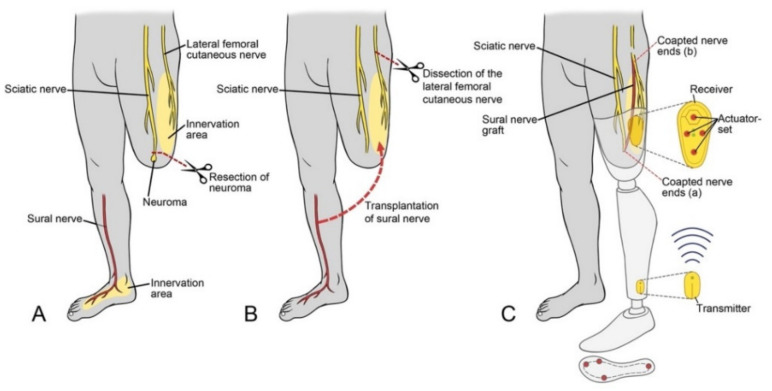
Illustration of the surgical technique for a transfemoral amputation: (**A**) resection of the stump neuroma; (**B**) transplantation of the contralateral sural nerve as an autologous graft from the distal stump of the sciatic nerve to the cutaneous territory of the lateral femoral cutaneous nerve; (**C**) the medical device Suralis in place, providing sensory feedback from the sole to the reinnervated skin area.

Through a dorsal approach, the bifurcation of the sciatic nerve is visualized, and the neuromas are identified and resected. The sural nerve with its origins from the tibial and common peroneal nerve is identified and followed distally, where its neuroma is resected as well. The sural nerve graft is then sutured to the distal sural nerve on the affected side. The prolonged sural nerve can now be brought up to the lateral thigh in a subcutaneous fashion. After repositioning the patient into the supine position, the lateral femoral cutaneous nerve is visualized just distal to the inguinal ligament, before its bifurcation. The nerve is transected under subtle tension far proximally in the lacuna musculorum below the inguinal ligament so that the proximal stump comes to lie cranially to the inguinal ligament in the pelvic cavity. As a result, the neuroma is not perceived symptomatically. Now the sural nerve, prolonged by the autologous graft, is coaptated to the distal stump in an end-to-end fashion. The surgical approach for trans-femoral amputees is depicted in Figure 3.

In a prophylactic setting (such as in patient 3), the sural nerve can be harvested from the affected leg over its entire course, thus rendering a contralateral graft unnecessary. 

### 2.6. Rehabilitation

After TSR surgery, donning of the prosthesis is discontinued for 6 weeks to avoid compromising the surgical incision as well as the nerve coaptations. Afterwards, all patients were fitted with a modified prosthesis equipped with a pressure sensor in the sole. The signals generated here are then transmitted to an actuator placed on the reinnervated skin area. This setup allows for the impulses coming from the prosthetic sole to be perceived as genuinely coming from the lost foot.

### 2.7. Physical Therapy

Physical therapy should begin immediately after surgery with local treatment of the residual limb: scar treatment, lymph drainage, bandaging, lymphatic tape, myofascial manual techniques. TENS therapy (transcutaneous electrical nerve stimulation) can be started after completion of wound healing (generally after 2–3 weeks) for 15 min twice a day. The electrical stimuli should be deep enough to stimulate and support the nerve fiber sprouting directly at the nerve for the reinnervation of the transposed or transplanted nerve. As soon as the first regenerating nerve fibers reach the skin, additional vibration therapy can be started. After the prosthesis is fitted, the sensory feedback system is installed as an add-on to the existing care with accompanying integrative physiotherapy, gait training, proprioceptive stimulation, fall prevention, and stair climbing.

## 3. Results

**Figure 4:**
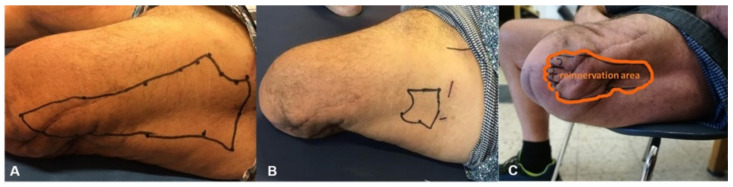
(**A**) Numb skin area 4 weeks after denervation; (**B**) size reduction of the numb skin area 9 months after TSR surgery; (**C**) patient’s perception of the foot within the surgically reinnervated skin area (patient 2).

Sensory reinnervation began 4 to 6 months after surgery. At the end of the follow-up, the initially numb skin area was significantly reduced in size in all patients (Figure 3A,B). All patients perceived a sensory image of their lost foot in the reinnervated skin area of the stump (Figure 4C, Table 2). 

**Figure 5:**
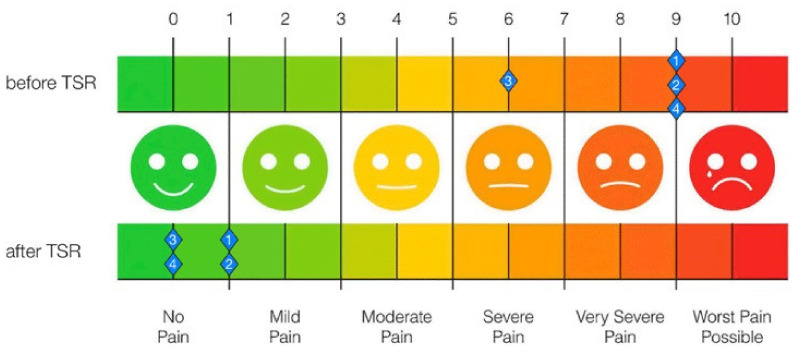
NRS scores before and after TSR surgery show a significant reduction in pain.

NRS scores were significantly reduced in all patients. Permanent pain medication could even be discontinued in all but one patient. A summary of these findings is given in Table 3 and Figure 5.

**Figure 6:**
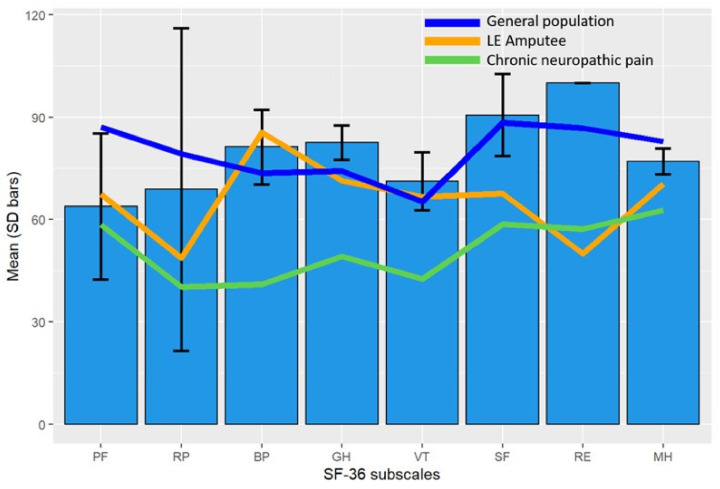
Comparison of mean test scores of the eight SF-36 subscales for TSR patients (blue bars) with the age-adjusted mean SF-36 scores of the general German population (navy blue line), lower extremity amputee population (orange line), and chronic neuropathic pain population (green line). Eight subscales measuring different domains of health-related quality of life: physical functioning (PF), role limitations—physical (RP), bodily pain (BP), general health (GH), vitality (VT), social functioning (SF), role limitations—emotional (RE), and mental health (MH). SD: Standard Deviation; LE: Lower Extremity.

Postoperative SF36 scores showed no significant difference compared with the general German population, except for general health. Compared with a lower extremity amputation and a chronic neuropathic pain population, TSR patients reported significant better scores in 4 respectively 6 subscales (Figure 6). 

All patients said they would undergo the surgery again. For one patient (patient 4), the comparison of four step square test, six-minute walk and timed up and go tests with a conventional prosthesis and a prosthesis providing vibro-tactile feedback on the reinnervated skin area was available. It showed significant functional advantages of the latter (Table 4).

## 4. Discussion

In amputees, a lack of sensory information from the missing limb leads to overcompensation with spontaneously generated signals in the brain and, consequently, to PLP. To this point, there is no causal treatment for PLP, and the symptomatic treatments applied are often insufficient to properly manage pain in these patients [6,28,29,30]. Amputees not only suffer from PLP, but also frequently experience postamputation NP, which precludes comfortable prosthesis wear [30,31]. NP can be prevented or treated by the surgical technique involving an epineural cap and transposition into muscle (Figure 1) or by prophylactic regenerative peripheral nerve interfaces in autologous free muscle grafts [32]. TMR is a surgical method by which an expendable muscle is surgically denervated and then reinnervated via its motor branch by another nerve. With this surgical intervention, symptomatic neuroma-related residual limb pain and pathologic phantom pain can be reduced both at the time of limb loss [33,34,35] and in secondary surgical intervention [30,31,36]. The disadvantage of TMR is that this method cannot provide sensory feedback to the amputee [11]. Sensory feedback can be restored, however, by means of invasive techniques or non-invasive techniques, such as electrotactile [37,38] and vibrotactile [39,40] stimulations [41]. Several reports have been published using implanted electrodes to stimulate the residual sciatic nerve with feedback from the prosthesis. For instance, Zelechowski et al. developed an anatomical computational model of the sensory stimulation for the sciatic nerve introducing sensory feedback by implanting transversal intrafascicular multichannel electrodes allowing amputees to feel touch sensations from a missing leg [24]. Another study using this method was published by Petrini et al. who reported that restoration of sensory feedback in lower limb amputees improved walking speed, metabolic demand, and phantom pain [23]. With regard to non-invasive techniques, Dietrich et al. performed sensory feedback using electrical stimulation. They mention that about one third of the patients had problems with the type of feedback and recommend the use of a more natural feedback such as vibrotactile feedback systems [21]. Crea et al. showed in their work that using vibrotactile units, the spatial and temporal relationship between the feedback system and the specific gait-phase transitions can be easily learned, but the main challenge is a system providing such feedback promoting the physiological gait pattern [20]. Not only TMR based methods but also these above-mentioned methods have the disadvantage that they do not target a specific reinnervated skin area. Thus, even if some kind of sensory feedback is evoked, it is not perceived as coming from the foot but rather from some other unphysiological area. These shortcomings can be solved by TSR, in which sensory nerves are diverted and used to transmit sensory information providing sensory feedback to the amputee that genuinely feels like originating from the lost limb. Thus, TSR might serve as a crucial step in the causal treatment of PLP by resupplying information to the brain. While the majority of amputations are performed on the lower extremity, to our knowledge, TSR has not yet been applied in this context, especially not as a first step in a holistic treatment plan incorporating a modified prosthetic device that is able to transfer sensory input from the prosthetic sole to the reinnervated skin area by vibrotactile stimulations. Therefore, we developed a TSR-based approach using and reactivating existing nerves. With this method, an area in the stump of the amputated lower limp is reactivated and reinnervated with existing autologous nerves (i.e., sural nerve) to give back the authentic feeling of the foot and reduce or prevent PLP as well as NP. Our method also allows for feedback from the prosthetic foot that is genuinely felt as if the lost foot was touched or brought into contact with the ground. This regained feedback from the periphery can then potentially be reintegrated into the cortical limb representation. Therefore, the brain no longer needs to compensate for this loss of signals by autonomously generated signals, potentially reducing PLP. Moreover, due to the improved feedback regarding the position of the prosthetic foot in space and its load bearing, prosthetic rehabilitation is facilitated, and functional parameters are improved. Given the high rate of PLP and NP in patients undergoing amputation, the technique for prophylactic TSR, as described here, could potentially be integrated into the treatment plan of amputees. This would eliminate the need for harvesting an autologous graft from the unaffected side and, moreover, reduce the duration of surgery.

In conclusion, TSR seems a reasonable option for the treatment of PLP and enabling interaction with bionic prostheses. By relocating the sural nerve to the saphenous nerve in transtibial or the lateral femoral cutaneous nerve in transfemoral amputees, the patients regain a feeling of the sole of the foot at the reinnervated area. In combination with a special prosthetic device and a comprehensive rehabilitations program, the newly reinnervated area on the thigh or lower leg receives sensory information from the prosthetic foot and could be defined as bionic reconstruction in the lower leg. Finally, the patient is able to feel his foot authentically, where it was. Physiological sensory information from the limb, which replaced the autonomously generated signals, results in a reduction or termination of PLP. Because of the retrospective nature of this study and the small number of patients, we were unable to account for all aspects of treatment, and only one patient had undergone gait assessment and analysis. Therefore, we think that a larger scale clinical trial on TSR for lower limb amputees with PLP seems warranted. 

## Figures and Tables

**Table 1:** Summary of patients and their pre-operative status.

Patient	Sex	Age at Injury (Year of Injury)	Mechanism of Injury	Side of Injury	Number of Operations	Age at Amputation (Year of Amputation)	Time between Injury and Amputation	Height of Amputation	Massive Phantom Limb and Neuroma Pain	Date of TSR Surgery	Donor Nerve	Target Nerve
1	Male	46 (2007)	Stroke and deep vein thrombosis	Left	4	46 (2007)	4 months	Posttraumatic Limb	Yes	October 2014	Ipsilateral sural nerve	Saphenous nerve
2	Male	25 (1989)	Motorcycle accident	Left	35	41 (2005)	16 years	Posttraumatic Thigh	Yes	November 2018	Contralateral sural nerve	Lateral femoral cutaneous nerve
3	Male	42 (2017)	Car accident	Left	12	44 (2019)	2 years	Elective Thigh	No	November 2019	Ipsilateral sural nerve	Lateral femoral cutaneous nerve
4	Female	50 (2011)	Motorcycle accident	Left	6	50 (2011)	1 day	Acute Limb	Yes	February 2020	Contralateral sural nerve	Lateral femoral cutaneous nerve

**Table 2:** Summary of post-operative results.

	TSR Operation	Begin of Physical Therapy	Type of Physical Therapy	Begin of Sensation	Sensation of the Foot on Reinnervated Skin Area	Phantom Limb Pain	Permanent Pain Medication	Status
1	October 2014	3 weeks after surgery	Tens and Vibro	5 months after surgery	Whole foot with toes, heel, Achilles tendon	No	Yes, for residual limb pain	Regeneration completed
2	November 2018	2 weeks after surgery	Tens and Vibro	6 months after surgery	Whole foot with toes, heel, Achilles tendon	No	None	Regeneration completed
3	November 2019	2 weeks after surgery	Tens and Vibro	4 months after surgery	Whole foot with toes, heel, Achilles tendon	No	None	Regeneration completed
4	February 2020	2 weeks after surgery	Tens and Vibro	5 months after surgery	heel, lateral part of the foot and foot sole, 4th and 5th toe, Achilles tendon	No	None	Regeneration in final phase

TSR: targeted sensory reinnervation.

**Table 3:** Pain medication before and after TSR.

Type of Medication	Before TSR [P#]	After TSR [P#]
Opioids	4, 3, 2, 1	1
Anticonvulsants	4, 2, 3, 1	0
NSAID	4	0
Novalminsulfon	4, 3	0
Antidepressant	1, 3	0
Cannabinoids	0	1
**Frequency of Medication**		
Nothing	0	2, 3, 4
on demand	0	1 (Opioids)
2–3 a day	2	1 (Cannabinoid)
4–5 a day	4, 3	
6 and more a day	1	

P#: Patient number; NSAID: Non-steroidal anti-inflammatory drug.

**Table 4:** Results for functional assessments (timed up and go, four square step and 6 min walk test) for patient 4 with conventional prosthesis and after 90 days with the medical device Suralis. Over all the tests, a significant functional improvement was seen.

	Conventional Prosthesis	Medical Device Suralis after 90 Days	Improvement in %
	Mean ± SD	Mean ± SD	Δ%
Timed Up the Go Test [s]	8.10 ± 0.31	7.44 ± 0.2	8.2%
Four Step Square Test [s]	10.03 ± 0.39	7.15 ± 0.28	40.1%
6 MWT [m]	450 ± 0	500 ± 0	11.1%

Δ%: Difference in percent between the measurements with conventional prosthesis and the Suralis device.

## Data Availability

All relevant data included in the study is given in this manuscript.
